# Determinants of Antimicrobial Resistance in *Acinetobacter baumannii* Isolates From Intensive Care Patients in Latvia

**DOI:** 10.1155/ijm/5526340

**Published:** 2026-03-28

**Authors:** Mihails Dolgusevs, Nityanand Jain, Oksana Savicka, Reinis Vangravs, Jevgenijs Bodrenko, Liene Ostele-Cešķe, Gustavs Rindžs, Dace Zemite, Aigars Reinis, Baiba Rozentale

**Affiliations:** ^1^ Department of Doctoral Studies, Riga Stradins University, 16 Dzirciema Street, Riga, LV-1007, Latvia, rsu.lv; ^2^ Intensive Care Unit, Liepaja Regional Hospital, 25 Slimnicas Street, Liepaja, LV-3414, Latvia; ^3^ Faculty of Medicine, Riga Stradins University, 16 Dzirciema Street, Riga, LV-1007, Latvia, rsu.lv; ^4^ Joint Microbiology Laboratory, Pauls Stradinš Clinical University Hospital, 13 Pilsonu Street, Riga, LV-1002, Latvia; ^5^ Department of Infectology, Riga Stradins University, 16 Dzirciema Street, Riga, LV-1007, Latvia, rsu.lv; ^6^ Laboratory “Latvian Centre of Infectious Diseases”, National Microbiology Reference Laboratory, Riga, LV-1006, Latvia; ^7^ Faculty of Residency, Riga Stradins University, 16 Dzirciema Street, Riga, LV-1007, Latvia, rsu.lv; ^8^ Laboratory “Centrala Laboratorija” Ltd., 25 Slimnicas Street, Liepaja, LV-3414, Latvia; ^9^ Department of Biology and Microbiology, Riga Stradins University, 16 Dzirciema Street, Riga, LV-1007, Latvia, rsu.lv; ^10^ Department of Public Health and Epidemiology, Riga Stradins University, 16 Dzirciema Street, Riga, LV-1007, Latvia, rsu.lv; ^11^ Latvian Centre of Infectious Diseases, Riga East Clinical University Hospital, Riga, LV-1006, Latvia

**Keywords:** *Acinetobacter baumannii*, genotype, intensive care units, phenotype, resistance

## Abstract

**Background:**

*Acinetobacter baumannii* is a leading nosocomial pathogen in intensive care units (ICUs), often resistant to multiple antibiotics. Data from the Baltic region remain scarce, limiting infection control and stewardship strategies.

**Methods:**

We conducted an integrated phenotypic–genotypic analysis of *A. baumannii* isolates collected from ICU patients in a tertiary‐care hospital in Latvia (July 2022–June 2024). Antimicrobial susceptibility testing was performed for major antibiotic classes, and whole‐genome sequencing (WGS) was used to identify genomic resistance determinants.

**Results:**

We analysed 52 clinical isolates from 45 ICU patients. Multidrug resistance was nearly universal (98%), with complete resistance to carbapenems and fluoroquinolones and > 95% resistance to aminoglycosides and trimethoprim–sulfamethoxazole. Colistin activity was largely preserved, with resistance detected in only one isolate, despite widespread polymyxin resistance–associated mutations. Genotypic findings were mostly in line with the phenotypic results. All isolates belonged to the ST2 lineage, highlighting clonal homogeneity. No plasmid replicons were detected, suggesting chromosomal elements as the primary resistance drivers.

**Conclusions:**

Our first integrated dataset in an ICU setting from the Baltic region demonstrates alarming resistance levels and clonal dominance of ST2. Our findings highlight the importance of combining WGS with susceptibility testing for accurate resistance assessment.

## 1. Introduction

*Acinetobacter baumannii* is a Gram‐negative, aerobic, nonfermenting coccobacillus that has emerged as a major opportunistic pathogen in healthcare settings [1, 2]. Recognized by the World Health Organization (WHO) as part of the ESKAPE group of pathogens, it often causes severe nosocomial infections, leading to high morbidity and mortality [1]. The clinical burden is greatest among vulnerable patients, especially those admitted to the intensive care units (ICUs), where prolonged hospitalization, use of invasive devices and immunosuppression substantially increase the risk of infection [2, 3].

The pathogen is remarkably resilient, with the capacity to survive on dry surfaces for extended periods, resist commonly used disinfectants and evade antimicrobial therapy, enabling its persistence and transmission within hospital environments [2, 3]. Over the past 2 decades, the global emergence of multidrug‐resistant (MDR) and extensively drug‐resistant (XDR) strains has severely limited our treatment options [4, 5]. Among them, the carbapenem‐resistant *A. baumannii* (CRAB) is of particular concern, with resistance typically mediated by class D OXA‐type carbapenemases (e.g. *blaOXA-23*), in combination with intrinsic *blaOXA-51*‐like genes and Acinetobacter‐derived cephalosporinases (ADCs) [6]. These findings have prompted the WHO to list CRAB as a ‘Critical Priority’ pathogen for the development of new antimicrobials [7].

Beyond *β*‐lactamase production, *A. baumannii* relies on multiple additional mechanisms of antimicrobial resistance (AMR), including aminoglycoside‐modifying enzymes (AMEs), target‐site mutations in *gyrA* and *parC* genes (against fluoroquinolones), sulphonamide resistance genes (*sul1, sul2*), alterations in lipopolysaccharide (LPS) biosynthesis (against colistin) and overexpression of efflux pump genes, such as *AdeABC, AdeIJK* and *AdeFGH* [8, 9]. The interplay of these mechanisms contributes to substantial phenotypic–genotypic heterogeneity, with whole‐genome sequencing (WGS) frequently revealing resistance genes that are not phenotypically expressed, and vice versa [10, 11]. Such discordance has important implications, as overreliance on a single method can lead to misinterpretation of resistance potential and affect clinical therapeutic decisions. Integrating both phenotypic and genotypic data is therefore essential for a more accurate characterization of resistance mechanisms and their clinical relevance.

In the Baltic region of Eastern Europe (Estonia, Latvia and Lithuania), molecular epidemiology of *A. baumannii* clinical isolates remains poorly characterized. Our team recently carried out the first national surveillance in Latvia which identified interhospital transmission of high‐risk clones, such as ST2 and ST570, carrying diverse carbapenemase and *β*‐lactamase gene profiles [12]. However, as this surveillance relied solely on WGS data and did not evaluate the phenotypic data, the findings were limited in their clinical applicability. This knowledge gap hampers evidence‐based infection control, outbreak detection and antimicrobial stewardship, while heightening the risk of silent dissemination of highly resistant clones across healthcare facilities nationwide. In fact, we have previously demonstrated the utility of such studies in the context of nosocomial *Pseudomonas aeruginosa* infections [13].

Hence, in this study, we characterized the first integrated phenotypic–genotypic data of *A. baumannii* isolates from ICU patients in a Latvian tertiary‐care hospital. We assessed antimicrobial susceptibility profiles alongside WGS‐derived resistance determinants, with emphasis on last‐line agents, such as colistin. Our findings aim to inform national infection control protocols, guide empirical therapy and place local Latvian data within a broader regional and global epidemiological context.

## 2. Materials and Methods

A prospective observational, single‐centre study was conducted at the Liepaja Regional Hospital in Latvia, a tertiary‐care facility with 349 beds and 10 ICU beds. The study protocol was approved by the Medical and Biomedical Research Ethics Committee of the Riga East University Hospital Support Foundation (vide N° 8‐A/22, dated 26.07.2022). Before participation and sample processing, patients or their relatives provided signed informed consent forms for inclusion of the clinical data in the study [13].

### 2.1. Inclusion Criteria

We collected *A. baumannii* nosocomial isolates from adult patients who were admitted to our ICU, irrespective of the diagnosis, from 1 July 2022 to 30 June 2024. All enrolled patients had a documented *A. baumannii* infection, as determined using standard laboratory microbiological testing either at the time of the ICU admission process or during the ICU stay. Patients not meeting these criteria or with a documented *A. baumannii* infection before transfer to the ICU were excluded from our analysis. We considered multiple samples from the same patient as separate isolates if there was a difference in the phenotypic resistance profile [13].

### 2.2. Clinical Assessment of Infection Versus Colonization

Classification of *A. baumannii* infection versus colonization was based on routine clinical judgement for admitted ICU patients. Clinical samples were collected only from patients with clinical suspicion of infection. The suspicion was guided by established clinical criteria, including compatible signs and symptoms (e.g. hyperthermia or hypothermia, leucocytosis or leukopenia, purulent secretions, worsening respiratory symptoms or organ function), relevant laboratory findings, supportive radiological evidence where applicable and the clinical decision to initiate or escalate targeted antimicrobial therapy.

While established European Centre for Disease Prevention and Control (ECDC) definitions for healthcare‐associated infections informed the clinical framework used by treating physicians, cases were not formally categorized according to ECDC surveillance case definitions. ECDC criteria are primarily intended for epidemiological surveillance rather than bedside diagnosis, and strict application of all diagnostic elements is not always feasible in the real‐world ICU setting. Patients were included in the study only when clinicians considered them clinically significant and consistent with active infection. Samples representing asymptomatic colonization were excluded. In cases of diagnostic uncertainty, continued clinical deterioration despite targeted antimicrobial therapy, supportive imaging findings or repeated isolation of *A*. *baumannii* isolates during follow‐up sampling contributed to classification as infection rather than colonization.

### 2.3. Sample Collection

Clinical samples were collected by trained and experienced ICU nurses or clinicians and sent for microbiological investigation to the laboratory in accordance with the local protocols. Specimens were processed according to the relevant EUCAST guidelines. We excluded samples with improper labelling and those with incomplete patient identifiers [13].

### 2.4. Microbiological Identification

The collected *A*. *baumannii* specimens were cultured on Columbia agar with 5% sheep blood (BD BBL Becton Dickinson GmbH, Germany, and Mast Diagnostica GmbH, Germany). Genus and species identification was carried out using bacteriological tests based on morphological, culture and biochemical characteristics, such as Gram staining, the VITEK‐2 analyser (BioMerieux, France) and the MALDI Biotyper (Bruker, Germany, USA) [13].

### 2.5. Phenotypic Susceptibility Testing

We used the agar disc diffusion method for antibiotic susceptibility testing. The Mueller–Hinton II Agar was used along with the European Committee on Antimicrobial Susceptibility Testing (EUCAST; Mast Diagnostica GmbH, Germany) and MASTDISCS AST (Mast Diagnostica GmbH, Germany) discs. Susceptibility results were interpreted according to the EUCAST guidelines for *A. baumannii*. Susceptibility testing was performed against the following antibiotics: amikacin, ciprofloxacin, gentamicin, imipenem, meropenem and trimethoprim/sulfamethoxazole (cotrimoxazole 1.25 μg/23.75 μg). Breakpoint tables for interpretation and zone diameters were based on v12.0 (from 1 January 2022 to 31 December 2022) [14], v13.1 (from 1 January 2023 to 31 December 2023) [15] and v14.0 (from 1 January 2024 onwards) [16]. For colistin, the minimum inhibitory concentrations (MICs) were determined using the VITEK‐2 analyser and re‐validated using the broth microdilution method (MICRONAUT MIC‐Strips, Bruker, Germany).

### 2.6. DNA Extraction and Concentration

DNA was extracted using the DNeasy Blood & Tissue Kit (QIAGEN, Germany), in accordance with the manufacturer’s protocol. DNA concentration was assessed with a Qubit 4 Fluorometer (Thermo Fisher Scientific, USA) and the Qubit dsDNA BR Assay Kit (Thermo Fisher Scientific, USA) [13].

### 2.7. Library Preparation and Sequencing

Libraries were prepared using the Illumina DNA Prep Kit (Illumina, USA) and then quantified with the Qubit dsDNA HS Assay Kit (Thermo Fisher Scientific, USA). Size distribution was estimated using TapeStation 4200 with the High Sensitivity D1000 ScreenTape (Agilent, Germany). Libraries were then pooled in equimolar proportions and further quantified using quantitative polymerase chain reaction (qPCR) with the Colibri Library Quantification Kit (Thermo Fisher Scientific, USA). The pooled libraries were sequenced on either the Illumina NextSeq 550dx or NovaSeq 6000 platforms (Illumina, USA) using 150PE or 250PE configurations [13]. Sequencing yielded a median of 7,713,806 paired‐end reads per sample (range: 1,809,452 to 23,536,300).

### 2.8. Bioinformatics Analysis

We used high‐performance computing cluster using an in‐house pipeline, Ardetype v1.0.0. [17]. Raw reads were filtered with Fastp v0.22.0 using default parameters, while human DNA contamination was removed using the GRCh38.p13 human reference genome with Kraken2 v2.1.2. [13, 18]. Filtered reads were assembled using Shovill v1.1.0 [19], and assemblies were evaluated with QUAST v5.0.2 using default parameters [20].

### 2.9. Resistance Gene Detection

AMR genes and point mutations were identified using the AMRFinderPlus v3.10.42 (database version 2022–10–11.2) [21], ResFinder v4.1.11 (database version 2023–03–29) [22] and RGI v5.2.1 (database version CARD v3.1.4) [23]. All tools were used with contigs and default parameters. ResFinder settings used included identity ≥ 0.6 and coverage ≥ 0.9; RGI settings were perfect and strict hits only with contigs > 20,000 base pairs (bp). Results from different databases were aggregated based on gene ontology to identify overlapping resistance genes [13].

### 2.10. Virulence Screening

Virulence determinant screening was performed on assembled genomes using VirulenceFinder (version dated 22 May 2025), with the corresponding virulence gene database version (dated 18 June 2024). Analyses were conducted on short‐read assemblies using default parameters.

### 2.11. Plasmid and Typing Analysis

Finally, PlasmidFinder v2023–03‐17 (database version 2024‐06–18) was used to detect plasmids [24]. Species were confirmed with Kraken2 and rMLST. Locus‐based typing included 7‐gene MLST v2.19.0 using PubMLST typing schemes [19, 25]; rMLST via PubMLST RESTful API [25]; and cgMLST using chewBBACA v3.1.2 [26] with *A. baumannii*‐specific scheme from cgmlst.org (Ridom, Germany, accessed 31 August 2024) [27, 28]. Minimum spanning tree was inferred from cgMLST data and visualized using GrapeTree v1.5.0 with the MSTreeV2 algorithm [29]. Core‐genome alignment was constructed by extracting allele sequences from genome assemblies based on chewBBACA‐inferred loci coordinates using BEDTools v2.31.0. Each locus was aligned individually with MAFFT v7.526 using the auto setting. The resulting alignments were concatenated into a single core‐genome alignment using Goalign v0.3.6. A maximum likelihood phylogeny was then inferred from the concatenated alignment using IQTREE v2.3.6, with automatic model selection enabled [13].

### 2.12. De‐Duplication of Isolated Genomes

To confirm the genomic distinctness of isolates and exclude potential duplicates (especially in cases of repeated samples from the same patient), a single nucleotide polymorphism (SNP)–based pairwise distance analysis was performed. Pairwise SNP distances were calculated from the concatenated core‐genome alignment using snp‐dists v0.8.2, generating a SNP distance matrix for all isolates. Distances between isolates were interpreted as the number of nucleotide differences across the shared core genome. Isolate pairs with a SNP distance of zero were considered to represent either potential duplication or extremely high genetic similarity, depending on the clinical sampling context.

### 2.13. Data Collection and Analyses

We collected the following clinical data from the electronic medical records: age, gender, reason for ICU admission, specimen type, sampling and testing completion date, duration of ICU stay, duration of hospital stay and final clinical outcome [13]. Therapeutically, we also extracted data on mechanical lung ventilation, continuous renal replacement therapy, antibiotic use and vasopressor use. All collected data were managed using MS Excel spreadsheets. Visualizations were prepared using MS Excel, custom Python scripts and R Studio v547dcf86 (build 2023–07‐07) for Windows 11.

## 3. Results

**FIGURE 1:**
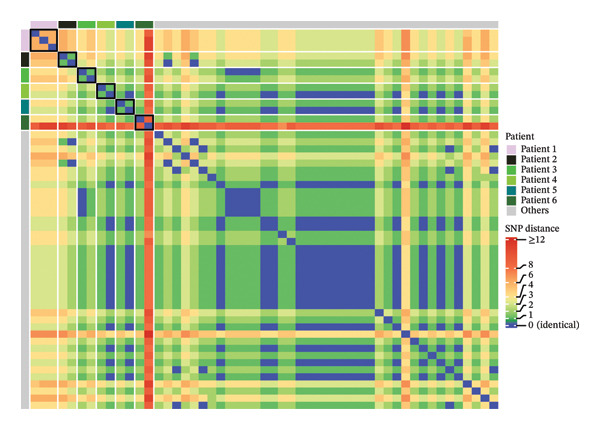
Pairwise SNP distance heatmap between all *Acinetobacter baumannii* isolates, calculated from a concatenated alignment of sequences from universally present cgMLST loci extracted from short‐read assemblies. Patients with multiple isolates are anonymized and highlighted (patients 1–6); all remaining patients are grouped as ‘others’ (grey). Isolates are ordered by patient group. Black boxes mark within‐patient comparisons for patients with multiple isolates (diagonal blocks). The colour scale represents SNP distance across the shared core alignment; 0 SNP (blue) indicates identical genomes, whereas higher values indicate increasing genetic divergence (distances ≥ 12 are capped at the maximum colour).

**FIGURE 2:**
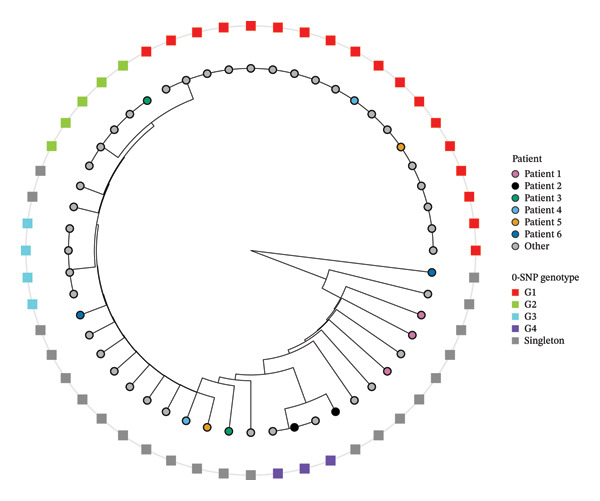
A circular dendrogram derived from the pairwise SNP distance matrix. Tips represent individual isolates. The outer ring (squares) denotes 0‐SNP genotypes (i.e. isolates identical across the analysed core‐genome alignment): Coloured squares (G1–G4) indicate multi‐isolate 0‐SNP genotypes, while grey squares indicate singleton genotypes. The inner ring (circles) indicates patients—multiple isolates from the six patients are shown in colour (patients 1–6), while isolates from all other patients are shown in grey (‘other’).

From 1049 patients admitted to our ICU between July 2022 and June 2024, a total of 2395 clinical samples were collected and tested. *A. baumannii* infection was confirmed in 52 samples (2% of all samples) from 45 patients (4% of all patients). To exclude potential duplicate genomes arising from repeated sampling from the same patients, we calculated pairwise SNP distances from a concatenated alignment of universally present cgMLST loci (Figure 1). All within‐patient pairs differed by at least 1 SNP (range 1–6), confirming the absence of duplication and supporting their treatment as independent observations in our analysis. Nonetheless, it is important to note that these differences may still represent closely related isolates within the same lineage. In contrast, several isolates from different patients were identical by our classification approach (0 SNP), consistent with circulation of shared genotypes across patients (Figure 2).

Demographically, more than half of the included patients were male (60%), and the median age of the cohort was 70 years (range 27–90 years). Bronchoalveolar lavage accounted for about 40% of the samples tested, followed by tracheal aspirate (13%) and sputum (10%). The median length of hospital stay was 27 days (range 4–124 days). Infection with *A. baumannii* was associated with poor clinical outcomes, with 76% of patients experiencing mortality and 20% requiring readmission to ICU. The median duration of ICU stay was 16 days with a range of 1–69 days (Table 1).

**TABLE 1:** Overview of the clinical data for 52 clinical isolates obtained from 45 patients admitted to ICU with *A. baumannii* infection.

Sample ID	Sex	Age (years)	Site of infection	Sample	Hospitalization days	No. of ICU days	Reason for ICU admission	Readmission to ICU	MPV	RRT	NE dosage	Other vasopressors	Co‐infection sepsis	Clinical outcome
8670278	M	79	GIT	FS	6	5	Cardiovascular	No	No	Yes	> 15	Dobutamine	None	Exitus letalis
6320471	F	53	GIT	FS	25	18	Pancreatitis	No	No	Yes	None	None	None	Discharged
2592861	M	86	Lungs	Sputum	12	10	Cardiovascular	No	Yes	No	> 15	Dobutamine	None	Exitus letalis
2651020	M	49	Lungs	Sputum	77	48	Trauma	Yes	Yes	Yes	> 15	Dobutamine and adrenaline	*E. faecalis* *and S. epidermidis*	Discharged
2651002	F	77	Lungs	BAL	16	10	CNS insult	No	Yes	Yes	> 15	Dopamine	None	Exitus letalis
**2628827**	F	62	Lungs	Sputum	19	17	CNS lymphoma	No	Yes	No	5 to 10	None	None	Exitus letalis
**2676044**														

2651240	M	76	Lungs	BAL	26	17	CNS hypertonic crisis	No	Yes	No	None	None	None	Exitus letalis
8817169	M	89	Lungs	BAL	7	4	Pneumonia	No	Yes	Yes	> 15	None	*S. hominis*	Exitus letalis
1249879	F	61	Wound	Wound	51	11	Pancreatitis	Yes	Yes	Yes	10 to 15	None	None	Exitus letalis
**3099859**	M	76	Lungs	Blood	67	37	Pulmonary	Yes	Yes	Yes	> 15	Dobutamine and empressin	*S. epidermidis*	Exitus letalis
**3238291**				FS										

3240048	F	67	Wound	Wound	16	12	CNS insult	No	Yes	No	> 15	None	*S. hominis*	Exitus letalis
3269460	M	75	GIT	FS	4	4	CNS insult	No	Yes	No	10 to 15	None	None	Exitus letalis
3408730	M	68	Blood	Blood	28	27	Pancreatitis	No	Yes	Yes	> 15	None	None	Exitus letalis
6384488	M	27	GIT	FS	11	11	Trauma	No	Yes	No	None	None	*S. epidermidis*	Discharged
7648435	M	80	Urinary	Urine	67	25	Aortic dissection	No	Yes	Yes	> 15	None	None	Discharged
3526395	F	76	Lungs	Sputum	26	9	CNS ICH	No	Yes	Yes	10 to 15	None	None	Exitus letalis
**7665435**	M	79	Lungs	BAL	73	69	Tick born encephalitis	Yes	Yes	No	> 15	None	*P. aeruginosa*	Exitus letalis
**7769541**														

7534990	M	33	Lungs	BAL	7	6	Sepsis	No	Yes	No	> 15	Dobutamine	*K. pneumoniae*	Exitus letalis
7572908	F	90	Lungs	TA	46	43	Trauma	No	Yes	No	5 to 10	None	None	Exitus letalis
**7609273**	M	53	Lungs	TA	33	28	CNS SDH	No	Yes	No	> 15	None	None	Exitus letalis
**7644436**			Urinary	Urine										

7609342	M	61	Lungs	TA	59	45	Anaphylactic shock	No	Yes	No	> 15	None	None	Exitus letalis
7644646	F	53	GIT	Drainage	31	1	Pancreatitis	No	No	No	None	None	None	Discharged
7773029	F	59	Lungs	BAL	43	31	CNS other	No	Yes	Yes	> 15	None	*S. epidermidis*	Exitus letalis
7670337	F	67	Lungs	BAL	5	5	CNS insult	No	Yes	Yes	> 15	Dobutamine	None	Exitus letalis
7772992	M	71	Lungs	BAL	16	16	CNS ICH	No	Yes	No	None	None	*S. epidermidis*	Exitus letalis
7852646	M	74	Lungs	TA	63	13	Cardiovascular	No	Yes	No	None	None	*S. haemolyticus*	Discharged
7892108	F	58	Lungs	TA	19	12	CNS insult	No	Yes	No	None	None	None	Exitus letalis
7904617	M	29	GIT	Punctate	8	5	Diabetes type I	No	Yes	No	< 5	None	None	Discharged
7943213	F	85	Lungs	BAL	9	9	CNS ICH	No	Yes	No	5 to 10	None	None	Exitus letalis
2318994	F	75	Wound	Wound	74	55	Sepsis	Yes	Yes	Yes	> 15	Adrenaline and dopamine	*S. haemolyticus and C. bifermentans*	Exitus letalis
8330701	M	39	Lungs	TA	71	33	Trauma (burns)	Yes	Yes	Yes	> 15	None	*E. cloacae*	Discharged
8363659	M	70	Lungs	BAL	16	10	CNS insult	Yes	Yes	No	5 to 10	Dopamine	None	Exitus letalis
8413270	F	73	Lungs	Blood	56	7	Cholecystitis and pancreatitis	Yes	Yes	Yes	> 15	None	*E. faecium*	Exitus letalis
**8458552**	M	86	Lungs	BAL	56	43	Erysipelas	No	Yes	Yes	> 15	Dobutamine and dopamine	*S. haemolyticus*	Exitus letalis
**8544277**			Lungs	BAL										
**8632497**			Urinary	Urine										

8452237	M	70	Lungs	BAL	43	40	Sepsis	No	Yes	Yes	> 15	Dobutamine	None	Exitus letalis
2342321	F	74	Lungs	BAL	19	14	CNS insult	No	Yes	Yes	None	None	None	Exitus letalis
8438654	M	54	Lungs	TA	27	17	Hypothermia	No	Yes	Yes	> 15	Dobutamine	None	Discharged
2355530	M	58	Lungs	Sputum	124	23	CNS insult	No	Yes	Yes	> 15	Dobutamine	None	Discharged
**8632425**	F	83	Urinary	Urine	87	16	Oesophageal tumour	Yes	Yes	No	> 15	Dobutamine	*S. marcescens*	Discharged
**8691566**	F													

2400875	F	77	Lungs	BAL	5	1	CNS insult	No	Yes	No	> 15	None	None	Exitus letalis
8626520	M	59	Lungs	BAL	25	19	CNS SDH	No	Yes	No	None	None	None	Exitus letalis
8650079	M	64	Lungs	BAL	28	16	CNS ICH	No	Yes	No	None	None	None	Exitus letalis
8619316	F	82	Lungs	BAL	13	12	Pneumonia	No	Yes	No	None	None	None	Exitus letalis
8680479	M	65	Lungs	BAL	34	28	CNS seizures	No	Yes	Yes	> 15	None	None	Exitus letalis
2562081	M	71	Lungs	BAL	28	28	CNS myxoedema coma	No	Yes	No	> 15	Dobutamine	None	Exitus letalis

*Note:* Nor‐epinephrine (NE) dosage units are mcg/min. Bold sample IDs indicate that more than one isolate was collected from the same patient. M: male; F: female; BAL: bronchoalveolar lavage fluid; ICH: intracerebral haemorrhage; SDH: subdural haematoma; GIT: gastrointestinal tract.

Abbreviations: CNS = central nervous system, FS = faecal screening, MPV = mechanical pulmonary (lung) ventilation, NE = nor‐epinephrine, RRT = renal replacement therapy, TA = tracheal aspirate.

### 3.1. Plasmid and Virulence Screening

Screening with PlasmidFinder detected no plasmid replicons in any of the included clinical isolates. Furthermore, screening for known virulence‐associated genes using VirulenceFinder did not identify any virulence markers in the analysed isolates. Importantly, screening using VirulenceFinder only allows for the detection of known virulence‐associated genes represented in the database but does not assess gene expression or regulatory activity.

### 3.2. Sequencing and Assembly Metrics

The sequencing data showed consistently high coverage across all isolates. After quality filtering, the average number of read pairs per sample was 8,772,292 (average read length: 204 bp), with a median of 7,254,526 read pairs (range: 1,785,672 to 22,934,830; median read length: 241 bp). The assembled genomes had an average and median length of approximately 4.1 Mb, indicating minimal variation in genome size. The average contig count was 71, with a median of 69, suggesting relatively contiguous assemblies. N50 values, indicative of assembly continuity, averaged 150,152 bp (median: 155,402 bp), reflecting high‐quality assemblies. Coverage depth was substantial, with an average of 468× and a median of 407×, supporting comprehensive genome representation. GC content was consistently 39.07% across samples, reflecting stable nucleotide composition.

### 3.3. Molecular Typing

Seven‐gene MLST using the Pasteur scheme assigned the ST2 sequence type to all our clinical isolates.

### 3.4. Resistance to Aminoglycosides

**FIGURE 3:**
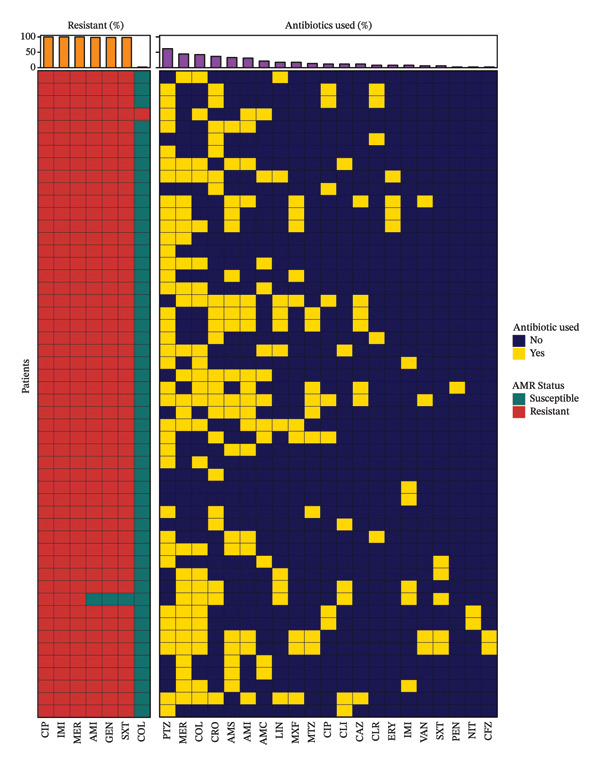
Antimicrobial resistance (AMR) and antibiotic usage patterns among nosocomial *Acinetobacter baumannii* isolates. Each row represents an individual isolate. Red tiles indicate AMR resistance, and green tiles indicate susceptibility. Yellow tiles denote administration of the antibiotic, and dark blue tiles indicate nonuse. Antibiotics on both heatmaps are arranged in descending order of resistance (left panel; orange bar chart) and usage frequency (right panel; purple bar chart), respectively. AMC—amoxicillin/clavulanate (amoxiclav); AMI—amikacin; AMS—ampicillin/sulbactam; CAZ—ceftazidime; CFZ—cefazolin; CIP—ciprofloxacin; CLI—clindamycin; CLR—clarithromycin; COL—colistin; CRO—ceftriaxone; ERY—erythromycin; GEN—gentamycin; IMI—imipenem/cilastatin; LIN—linezolid; MER—meropenem; MTZ—metronidazole; MXF—moxifloxacin; NIT—nitrofurantoin; PEN—penicillin G; PTZ—piperacillin/tazobactam; SXT—trimethoprim/sulfamethoxazole; VAN—vancomycin.

Phenotypic resistance to aminoglycosides was found to be nearly universal, with 98% of the isolates showing resistance to both amikacin and gentamicin and only a single isolate remaining susceptible (Figure 3; Table 2). Genotypic analysis corroborated these findings, revealing a high prevalence of AME genes. Notably, *aph(3″)-Ib* was detected in 100% of isolates, while other key AME genes including *ANT(3′)-IIc*, *APH(6)-Id*, *ant(3″)-IIa* and multiple *aac(6′)* variants were present in over 94% of cases (Figure 3). The single isolate that demonstrated phenotypic susceptibility to both amikacin and gentamicin notably lacked several high‐impact AME genes, including *aac(6′)* variants, *aadA1* and the 16S rRNA methyltransferase *armA*. However, it did retain a limited set of aminoglycoside resistance determinants, such as *ANT(3′)-IIc*, *APH(6)-Id*, *ant(3″)-IIa* and *aph(3″)-Ib*.

**TABLE 2:** Summary of antimicrobial susceptibility and clinical breakpoints for *A. baumannii* (*n* = 52).

Antibiotic	Method	Disc content (μg)	Range (min–max)	50th percentile^∗^	90th percentile^∗∗^	EUCAST breakpoint^∗∗∗^
Amikacin	Disc diffusion	30	6–25 mm	6 mm	6 mm	*R* < 19 mm
Ciprofloxacin	Disc diffusion	5	6‐6 mm	6 mm	6 mm	*R* < 21 mm
Gentamicin	Disc diffusion	10	6–23 mm	6 mm	6 mm	*R* < 17 mm
Imipenem	Disc diffusion	10	6‐6 mm	6 mm	6 mm	*R* < 21 mm
Meropenem	Disc diffusion	10	6‐6 mm	6 mm	6 mm	*R* < 15 mm
Trimethoprim/sulfamethoxazole	Disc diffusion	1.25‐23.75	6–24 mm	6 mm	6 mm	*R* < 11 mm
Colistin	Broth microdilution	—	0.5–4 mg/L	1.5 mg/L	2 mg/L	*R* > 2 mg/L

^∗^50th percentile corresponds to ZD50 or MIC50 based on the method used.

^∗∗^90th percentile corresponds to ZD90 or MIC90 based on the method used.

^∗∗∗^The breakpoints were similar across all versions of EUCAST guidelines during the study reference period.

### 3.5. Resistance to Fluoroquinolones

**FIGURE 4:**
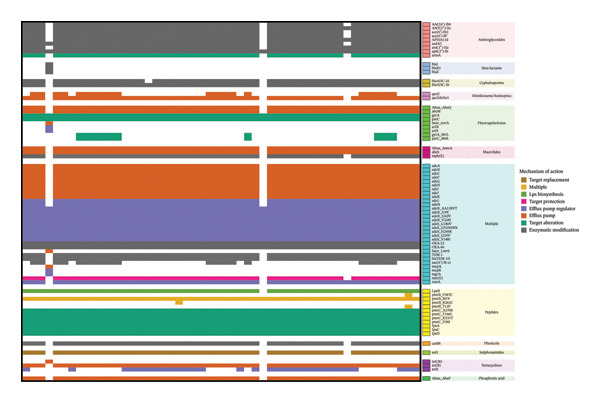
Heatmap of different resistance genes detected in the clinical isolates grouped by drug class of action. Each row represents a resistance gene (*n* = 77), and each column corresponds to an individual patient isolate (*n* = 52). The presence of a gene is indicated by a coloured cell, with colours assigned according to the gene’s primary mechanism of action. Data on drug class and mechanism of action were curated from the comprehensive antibiotic resistance database (CARD).

All isolates demonstrated phenotypic resistance to ciprofloxacin. Genotypic screening confirmed the universal presence of *gyrA* and *parC* genes, encoding the DNA gyrase and topoisomerase IV subunits, respectively. Notably, resistance‐associated point mutations, *gyrA_S81L* and *parC_S84L*, were detected in 10 isolates (19%; Figure 4).

### 3.6. Resistance to Carbapenems

Carbapenem resistance was absolute, with no isolate demonstrating phenotypic susceptibility to either imipenem or meropenem (Figure 3). Genotypic analysis identified the widespread presence of *OXA-23* and *OXA-66* genes—class D *β*‐lactamases known to mediate carbapenem hydrolysis. In addition, nearly all isolates harboured either *blaADC-25* (94%) or *blaADC-30* (96%) genes encoding ADCs which may further contribute to resistance against a broader spectrum of *β*‐lactam agents. The co‐occurrence of OXA‐type carbapenemases and ADCs provides a compelling molecular basis for the observed carbapenem resistance rates.

### 3.7. Resistance to Trimethoprim/Sulfamethoxazole

Phenotypic resistance to trimethoprim/sulfamethoxazole was observed in 98% of isolates, with only a single isolate remaining susceptible. Genotypic findings were concordant. The *sul1* gene, encoding a sulphonamide‐resistant dihydropteroate synthase, was identified in 49 of 52 isolates (94%; Figure 4). Interestingly, we did not detect *dfrA* or *dfrB* gene variants which typically mediate trimethoprim resistance.

### 3.8. Resistance to Polymyxins

Colistin activity was largely preserved phenotypically, i.e. resistance was identified only in a single isolate (defined as MIC > 2 mg/L), which was confirmed by broth microdilution. Nonetheless, genotypic analysis identified a high prevalence of mutations associated with polymyxin resistance (Figure 4). All isolates carried mutations in *pmrB* and *pmrC* genes, including *pmrB_M1V* and multiple pmrC variants (*A370S, F166L, K531T and V58I*), which have been implicated in colistin resistance through modulation of LPS structure and charge. Interestingly, only one isolate carried additional rare mutations, such as *pmrB_F387C*, *pmrB_R263C* and *pmrB_T13P*, as well as deletions or disruptions in *lpxA*, *lpxC* and *lpxD* genes, all of which are known contributors to LPS loss and high levels of colistin resistance.

### 3.9. Multidrug Resistance

Based on phenotypic susceptibility testing and applying the standardized definitions proposed by Magiorakos et al., as adopted by subsequent global surveillance frameworks including WHO’s Global Antimicrobial Resistance and Use Surveillance System (GLASS) [30, 31], 96% (50/52) of isolates fulfilled the criteria for XDR *A. baumannii*, reflecting nonsusceptibility to all but one or two antimicrobial categories. One isolate (2%) met the criteria for MDR but not XDR. Similarly, a single isolate (2%) was classified as having pan‐drug resistance (PDR), as susceptibility to colistin was preserved in all but one isolate. These findings underscore the critical therapeutic limitations in the treatment of nosocomial *A. baumannii* infections in contemporary intensive care practice.

## 4. Discussion

This study presents an integrated phenotypic–genotypic characterization of *A*. *baumannii* isolates from ICU patients in a Latvian tertiary‐care hospital, representing the first dataset of its kind in the Baltic region. Our results emphasize the need for continued genomic surveillance, timely incorporation of laboratory data into clinical decision‐making and tailored infection control interventions. The presence of high‐risk clones, extensive genomic resistance determinants and the vulnerability of critically ill patients in ICUs creates a setting in which treatment options are limited and containment becomes increasingly challenging.

Clinically, our cohort had characteristics consistent with those described elsewhere in the literature. Nearly two‐thirds of our patients had pulmonary infections, in line with the well‐documented role of *A. baumannii* as a major causative agent of ventilator‐associated pneumonia (VAP). Among European ICUs, carbapenem‐resistant strains are commonly isolated in VAP episodes [32, 33]. Pooled estimates for *A. baumannii-*associated VAP and other nosocomial infections have shown an increase of about 15% in the prevalence of MDR isolates, reaching 100% in certain high‐burden European and American regions [34]. Apart from pulmonary disease, *A*. *baumannii* is known to be a significant contributor of nosocomial bloodstream, wound and surgical site infections [35]. Though urinary and gastrointestinal infections are infrequent, they remain clinically relevant, especially in the context of polymicrobial infections [36, 37].

Notably, all isolates in our study belonged to the ST2 sequence type, which is strongly associated with VAP, bloodstream infections and wound infections. ST2 is among the most widely disseminated *A. baumannii* lineages in Europe and is recognized as a high‐risk clone due to its MDR, adaptability to hospital environments and strong outbreak potential [38, 39]. The marked clonal uniformity observed in our cohort suggests a high degree of relatedness among isolates. As we did not perform temporal and epidemiological linkage studies, definitive conclusions regarding clonal persistence, transmission dynamics, environmental reservoirs or infection control breaches cannot be drawn.

However, to provide external genomic context, representative isolates from our study were compared against publicly available *A. baumannii* genomes using cgMLST. Each selected isolate sequence differed by at least 35 alleles from the closest publicly available isolate, which exceeds commonly used allele‐distance thresholds for likely unrelated strains [40]. This confirmed that while the study isolates are closely related to each other (Figures 1 and 2), they remain genetically distinct from most previously published ST2 isolates [40]. Accordingly, interpretations regarding persistence or transmission of a dominant clone are restricted only to the study cohort and warrant further confirmation through longitudinal, multicentre studies incorporating detailed epidemiological data.

Irrespective of the epidemiological considerations, the absence of plasmid replicons in all isolates provides insight into the genomic architecture underlying AMR in our cohort. Large‐scale genomic studies have shown that a substantial proportion of *A. baumannii* isolates lack detectable plasmids. For instance, a survey of 450 complete genomes found that around 21% were plasmid‐free in the GenBank database [41], while pan‐genome analyses identified isolate groups that rarely harbour plasmids, often carrying CRISPR/Cas or restriction–modification systems that may limit plasmid acquisition [42]. In such cases, resistance may be predominantly maintained through chromosomal elements, such as *AbaR* islands and transposons. In our context, this likely reflects the genomic strategy of the ST2 lineage, which relies heavily on stable chromosomally encoded resistance determinants, including *blaOXA-23* and *blaADC* variants [38]. Clinically, such genomic architecture limits therapeutic options to last‐line agents, such as colistin or newer agents, such as cefiderocol [43], though emerging resistance to both underscores the urgency of effective infection control and antimicrobial stewardship.

Next, we observed a near‐universal resistance to aminoglycosides (amikacin and gentamicin) which was genomically strongly correlated with the presence of AMEs, particularly *aph(3″)-Ib*, *ANT(3′)-IIc*, *APH(6)-Id* and *aac(6′)* variants. This pattern aligns with reports from European and Asian hospital settings, where resistance rates frequently exceed 80%–90% and AMEs are identified as the predominant resistance determinants [44, 45]. The strong correlation between AME gene prevalence and phenotypic resistance suggests that cumulative or synergistic activity of these enzymes underpins aminoglycoside resistance observed in clinical isolates [45], rendering these drugs largely ineffective as monotherapy. However, despite retaining a set of AMEs, the only aminoglycoside‐susceptible isolate shows that certain classes of AMEs alone may not be sufficient to confer pan‐aminoglycoside resistance. We believe that clinical isolates likely require the cumulative activity of multiple AMEs, particularly those mediating acetylation and ribosomal methylation. Furthermore, studies have indicated that isolates with both AMEs and 16S rRNA methyltransferases, such as *armA*, consistently exhibit higher levels of resistance to all aminoglycosides [46, 47].

We also detected complete phenotypic resistance to ciprofloxacin. In 19% of our isolates, resistance‐associated target‐site mutations (*gyrA_S81L* and *parC_S84L*) were identified, reflecting the well‐established role of quinolone resistance–determining region (QRDR) alterations in conferring fluoroquinolone resistance. These substitutions impair drug binding to DNA gyrase and topoisomerase IV, and their presence has been linked with enhanced resistance in clinical isolates [48]. The presence of these mutations in a subset of strains suggests that while efflux and other mechanisms may underlie baseline resistance, target‐site alterations amplify resistance severity in a distinct subset of isolates.

At the same time, it is interesting that phenotypically resistance remained universal even among those isolates that lacked these point mutations and harboured the wild‐type alleles at these loci. In such isolates, resistance may have been likely mediated by alternative mechanisms, such as overexpression of efflux pumps (e.g. *AdeABC, AdeIJK* and *AdeFGH*) [8], or uncharacterized mutations beyond the scope of the current screening panel. Although no plasmid replicons or known plasmid‐mediated quinolone resistance (PMQR) determinants were detected in our samples, plasmid carriage cannot be definitively excluded given our use of only short‐read sequencing and replicon‐based detection methods. Nevertheless, the available genomic data support the interpretation that fluoroquinolone resistance in our samples may be predominantly driven by chromosomal mechanisms rather than acquired plasmid‐mediated determinants.

Regarding CRAB isolates, our observations are consistent with global molecular epidemiology trends, where carbapenem resistance among ICU isolates has been reported to be around 80%–100% [49, 50]. In our cohort, resistance was primarily mediated by *OXA-23* carbapenemases in combination with intrinsic *OXA-66* (*OXA-51*‐like) enzymes, accompanied by *ADC-25* or *ADC-30*. Similar resistance profiles have been reported in Europe [6, 7], Asia [51] and Latin America [52], confirming the global dominance of these *β*‐lactamases as key resistance determinants. The widespread distribution of *blaOXA-23* is particularly concerning given its frequent association with mobile genetic elements that promote horizontal gene transfer [6, 53]. The consistent detection of *OXA-66*, a variant of the intrinsic *OXA-51*‐like family, further highlights its conserved role in the resistance landscape. Although *OXA-66* alone is typically insufficient to confer clinically relevant resistance, its activity may be potentiated through overexpression or in tandem with acquired enzymes, such as *OXA-23*.

The near‐universal resistance to trimethoprim/sulfamethoxazole in our isolates was strongly associated with the presence of the *sul1* gene, which encodes an alternative dihydropteroate synthase with reduced affinity for sulphonamides. *Sul1* is frequently embedded within class 1 integrons and mobile genetic elements, facilitating its horizontal dissemination and persistence in clinical settings [54, 55]. The absence of *dfrA* or *dfrB* variants in our cohort is noteworthy, as these genes usually mediate trimethoprim resistance in Gram‐negative pathogens. This suggests that resistance to the trimethoprim component may instead be mediated by alternative mechanisms, such as mutations in the native dihydrofolate reductase or overexpression of efflux pumps, such as *AdeABC, AdeFGH* and *AbeM* [55].

The phenotypic susceptibility to colistin was preserved in 98% of isolates, despite the high prevalence of polymyxin resistance‐associated mutations (*pmrB, pmrC and lpxA/C/D*). This genotype–phenotype discordance has been reported elsewhere [56, 57] and may be due to low‐level or nonexpressive mutations that require additional regulatory changes for full resistance expression. These findings highlight the complexity of predicting polymyxin resistance genomically, reinforce the need of broth microdilution for resistance determination and caution against overinterpreting the presence of *pmr* variants in surveillance data.

Clearly, the observed inconsistencies between genotypic and phenotypic resistance profiles for different antibiotics underscore the multifactorial nature of *A. baumannii* resistance expression. Our findings highlight that the detection of a resistance gene does not always translate into clinical resistance and vice versa. This has significant implications for both clinical microbiology surveillance and antimicrobial stewardship: Reliance solely on WGS for therapeutic decisions risks overestimating resistance, as some detected genes may be transcriptionally silent or functionally inactive under current conditions. Conversely, phenotypic testing alone may miss latent resistance potential, as certain genes, although not expressed at the time of testing, can be active during treatment in response to selective pressure, regulatory mutations or environmental triggers. Such activation can occur rapidly, leading to the emergence of phenotypic resistance during treatment and potential therapeutic failure. Hence, it is crucial that phenotypic data are integrated with genotypic WGS data to provide a complete and clinically relevant resistance profile for guiding antimicrobial therapy.

### 4.1. Limitations and Future Directions

Despite the importance of our findings, there are several limitations that should be acknowledged. As a single‐centre study, the findings may have limited generalizability across other institutions across Latvia and/or the wider Nordic‐Baltic region. Second, the short‐read sequencing approach used herein could not fully resolve plasmid structures or the genomic context of mobile genetic elements carrying resistance genes, thereby limiting precise assignment of resistance determinants to chromosomal or plasmid locations. Moreover, we performed plasmid detection for known plasmid replicons using PlasmidFinder, applied to short‐read assemblies. This approach is inherently restricted to plasmids carrying replicons represented in the database and cannot reliably identify novel, highly divergent or cryptic plasmids, nor does it allow to reconstruct complete plasmid structures.

Future work would benefit from incorporating long‐read or hybrid sequencing approaches, which enable contiguous assembly of plasmids and mobile elements while allowing for more accurate reconstruction of resistance gene architecture [58, 59]. Hybrid assemblies have been shown to recover small plasmids and provide both structural resolution and base‐level accuracy [59] and could enhance characterization of plasmid‐mediated resistance in future studies. Another key limitation is the nonvalidation of gene expression using transcriptomic profiling or targeted qPCR especially for major resistance determinants including *pmrB*, *pmrC*, *sul1* and efflux pump systems (*adeABC*, *adeIJK* and *adeFGH*). Consequently, mechanistic interpretation of the observed genotype–phenotype discordance remains largely theoretical and indicative in nature. Nonetheless, our findings carry clinical significance and have real‐world implications. The observed discordance reflects on‐ground clinico‐microbiological diagnostic challenges and reinforces the necessity of phenotypic susceptibility testing alongside WGS, especially for antibiotics where resistance expression is strongly influenced by regulatory and environmental factors.

We also acknowledge that infection–colonization differentiation in our study relied on expert clinical judgement rather than formal surveillance case definitions. While this could have introduced some degree of subjectivity and misclassification risk, it helped us to better align with the clinical decision‐making triages, ensuring the clinical relevance of our findings. Next, although clinical outcomes, such as mortality, length of ICU stay and antibiotic exposure, were collected, this study was not designed or powered to support inferential analyses linking these outcomes to AMR phenotypes or specific genomic determinants. Resistance profiles were highly homogeneous across the cohort, and clinical outcomes in critically ill ICU patients are influenced by multiple confounding factors, including underlying comorbidities, disease severity and supportive care measures. Accordingly, clinical data are presented only in a descriptive manner to better contextualize disease burden rather than to establish genotype–phenotype–outcome associations.

Finally, clinical data on prior antibiotic exposures were not systematically collected, limiting our ability to assess antibiotic‐driven selection pressures. Though we present the data on antibiotic prescription for reference purposes (Figure 3), we could not reliably correlate their timeline with the phenotypic and genotypic resistance profiles. As such, data about antibiotic prescription should not be interpreted as evidence of causal relationships. Future prospective studies incorporating larger and more heterogeneous cohorts, structured treatment timelines and multivariable analytical frameworks will be required to robustly evaluate associations between AMR, genomic features and clinical outcomes.

## 5. Conclusions

We observed extremely high rates of MDR isolates in our cohort, with universal carbapenem resistance and near‐complete resistance to aminoglycosides, fluoroquinolones and trimethoprim/sulfamethoxazole. Colistin activity remained largely preserved, despite widespread presence of resistance–associated mutations. Our findings highlight the need for combining WGS and phenotypic susceptibility testing to capture the full spectrum of the resistome, with neither approach alone offering a complete assessment. Broader multicentre surveillance, integration of long‐read sequencing to resolve mobile genetic elements and linking molecular determinants to clinical outcomes will be needed in future to stem nosocomial spread and improve critical care prognosis.

## Author Contributions

Mihails Dolgusevs, Aigars Reinis and Baiba Rozentale conceptualized this study and led the investigations. Liene Ostele‐Cešķe, Gustavs Rindžs and Mihails Dolgusevs were responsible for clinical data collection and curation, while Dace Zemite, Jevgenijs Bodrenko, Oksana Savicka and Reinis Vangravs were responsible for laboratory analysis and data collection. Mihails Dolgusevs and Nityanand Jain validated the data and were involved in formal analysis. Nityanand Jain provided software and was involved in visualization. Nityanand Jain, Mihails Dolgusevs and Reinis Vangravs drafted the initial version of the manuscript. All authors were responsible for revising the manuscript. Mihails Dolgusevs and Oksana Savicka were involved in project administration and resource management, under the supervision of Aigars Reinis and Baiba Rozentale.

## Funding

This study did not receive any funding.

## Disclosure

All authors have read and agreed to the final version for publication. This study was conducted under the purview of doctoral work for Mihails Dolgusevs at Riga Stradinš University, Riga, Latvia.

## Ethics Statement

This study was approved by the Medical and Biomedical Research Ethics Committee of the Riga East University Hospital Support Foundation (N^o^ 8‐A/22, dated 26.07.2022).

## Conflicts of Interest

The authors declare no conflicts of interest.

## Data Availability

Data generated in this study are presented within the results section of the paper. Sequencing data have been deposited to and made publicly available at the European Nucleotide Archive (ENA) vide project accession no. PRJEB96155, dated 19 August 2025. Data can be accessed at https://www.ebi.ac.uk/ena/browser/view/PRJEB96155. Further queries and data requests can be directed to the lead study author Mihails Dolgusevs (mihails.dolgusevs@rsu.lv).
